# The spread of *Carpophilus truncatus* is on the razor's edge between an outbreak and a pest invasion

**DOI:** 10.1038/s41598-022-23520-2

**Published:** 2022-11-07

**Authors:** Flavia de Benedetta, Simona Gargiulo, Fortuna Miele, Laura Figlioli, Michele Innangi, Paolo Audisio, Francesco Nugnes, Umberto Bernardo

**Affiliations:** 1 Institute for Sustainable Plant Protection - IPSP-CNR, National Research Council, P.le E. Fermi, 1, 80055 Portici, NA Italy; 2 Department of Agricultural Sciences, University of Napoli Federico II, Via Università, 100, 80055 Portici, NA Italy; 3 Department of Biosciences and Territory, University of Molise, Contrada Fonte Lappone, 86090 Pesche, IS Italy; 4 Department of Biology and Biotechnologies “C. Darwin”, “Sapienza” University of Rome, Via A. Borelli, 50, 00161 Rome, Italy

**Keywords:** Invasive species, Entomology

## Abstract

In 2019, in southern Italy (Campania) there was an outbreak of a sap beetle infesting stored walnut fruits. A monitoring activity started to assess the spread and impact of the pest in walnut orchards and in warehouses, and an integrative characterization led to identify the beetle as *Carpophilus truncatus*. This species has been in Europe for a long time, rare and harmless until recently. We show also that this species is the same recently recorded in other two continents, Latin America and Australia, where it is causing massive damage on walnut and almond fruits. The sharing of a mitochondrial haplotype among populations recorded on three continents suggests that a worldwide invasion might be ongoing. A Geographic Profiling approach has determined that the more virulent population was first introduced in Italy, and the climate conditions of areas where *C. truncatus* is currently widespread and harmful indicate that the entire walnuts world production is in jeopardy as this species could adapt to any of the main walnut and almond production areas.

## Introduction

Invasive insects and pathogens represent an increasing threat to agriculture and forestry worldwide^1,2^. Despite the implemented preventative measures, the transport of goods (stored products, living plants and fruits), and people facilitate the introduction of invasive species in new areas^3,4^. And climate change promotes modification in the distribution range of pests and can cause the weakening of host plants and a higher level of damage by pests^5,6^. In some cases, even a newly introduced population of an already present species may represent a threat, due to peculiar biological traits or eventual specialization or different adaptation^7,8^. Genetic studies of introduced populations can provide helpful information about the colonization processes, enabling the reconstruction of the route of invasion and possibly highlighting differences with the native ones^9^. Usually, introduced populations have poor genetic diversity due to bottleneck and founder effects^10^. However, despite the low genetic variability, the populations/species may still be able to efficiently colonize new areas, a phenomenon known as the ‘genetic paradox’^11^. A rapid identification of species potentially invasive is the first and essential step to implement adequate containment measures. Morphological identification is often time-consuming and requires highly specialized knowledge^12^. Furthermore, phenotypic plasticity can result in high intraspecific variability, making the identification hardly unambiguous^12–15^. An integrative approach, including DNA barcoding and morphological methodology, can be the best technique to identify non-native species^16,17^.

The walnut ecosystem (*Juglans* spp.) in Italy has been recently impacted by the arrival and establishment of several invasive pests such as *Rhagoletis completa* Cresson (Diptera: Tephritidae), *Coptodisca lucifluella* (Clemens) (Lepidoptera: Heliozelidae), and *Pityophthorus juglandis* Blackman (Coleoptera: Curculionidae)^6–20^. In 2019, inspective activities in a walnut warehouse led to the discovery of many sap beetles (larvae and adults) on stored walnuts, identified morphologically by us as belonging to the genus *Carpophilus* Stephens (Coleoptera: Nitidulidae)^21^. This genus includes more than 280 species, many of which now with a worldwide distribution, but native mainly to tropical and subtropical regions^22–24^, with some species being serious pests^25^. About fifteen *Carpophilus* species are present in Europe and in the Mediterranean basin, including Italy, mostly introduced through the trade of foodstuffs and subsequently acclimatized over the last 2–3 centuries^23,26^. Most species are phyto-saprophagous (they feed on decaying vegetable substances) and develop on fruits and other organic substrates as larvae. *Carpophilus* beetles mostly attack ripe and ripening fruit, but also stone fruits, cereals and dried fruit, sometimes causing serious damage to crops also by transmitting yeasts and bacterial pathogens^23,27^. Recently, there have been two reports of unprecedented massive damage by *Carpophilus* on stored walnuts in Argentina^28^ and almonds in Australia^29–31^.

Our first attempt to identify the *Carpophilus* species most frequently recorded during our samplings was not fully resolutive^21^, due to the uncertain taxonomy of the genus^32,33^. Some species of the *C. dimidiatus* complex (within the *Myothorax* subgenus) are, in fact, very difficult to distinguish by morphology alone^24^. For this reason, it is indispensable to integrate the morphological observation with a molecular approach. Unfortunately, only a few species of *Carpophilus* are already genetically characterized, with a rather small number of sequences available in the Genbank and BOLD databases. In some cases, very different sequences are associated with the same taxon, creating confusion and possible misidentification. *Myothorax* species are particularly challenging in this respect and clearly need a revision^30^.

This study aimed at: a) providing a correct identification thorough characterization of the recorded species with an integrative approach (morphological and molecular); b) assessing population-level diversity to identify the most likely point of introduction; c) carrying out a risk assessment based on the type and intensity of the damage and potential diffusion based on the current diffusion. We have also clarified some aspects of the biology of this pest.

## Materials and methods

### Monitoring activities

Sampling activity was carried out in 2019 and 2020 from October to December in the Campania region (southern Italy). Monitoring was conducted in dried fruit warehouses, specialized and non-specialized walnut orchards (*Juglans regia* L.), and farms with walnut orchards and storage rooms. In each warehouse, twenty walnut fruits were randomly collected, and categorized as follows: “sorted” included walnut fruits stored in warehouses that have undergone mechanical and/or manual sorting process; “unsorted” included walnut fruits stored in a warehouse waiting to be sorted; “discarded” included walnut fruits discarded during the sorting process. In each walnut field, 20 fruits were randomly collected, picking up four fruits from the ground under five trees. Each walnut tree was located at least 50 m from each other. Twenty and thirty fruits were collected from each farm, from the ground and from each category of stored walnuts in 2019 and 2020, respectively. Sampled fruits were contained in double plastic bags to prevent insects from escaping, labeled, brought to the laboratory of the Italian National Research Council (CNR)—Institute for Sustainable Plant Protection (IPSP) in Portici (Naples, Italy) and stored at 4 °C until analysis. *Carpophilus* specimens were collected from areas that had been invaded by this species in Italy. The fruits of *Juglans regia* used in this study were collected under the permission of owners and following Italian and international rules and regulations. Experimental research and field studies on plants were carried out in accordance with relevant institutional, national, and international guidelines and legislation.

### Damage

Every sampled fruit was first inspected externally, to check for the presence of holes. After external observations, the shells were broken and insect presence, kernel damage and rotting symptoms were recorded. Adults and larvae of sap beetles (and moths, if present) were collected, counted, and placed in labeled Eppendorf tubes in 100% ethanol and stored at − 20 °C.

### Morphological characterization

**Figure 1:**
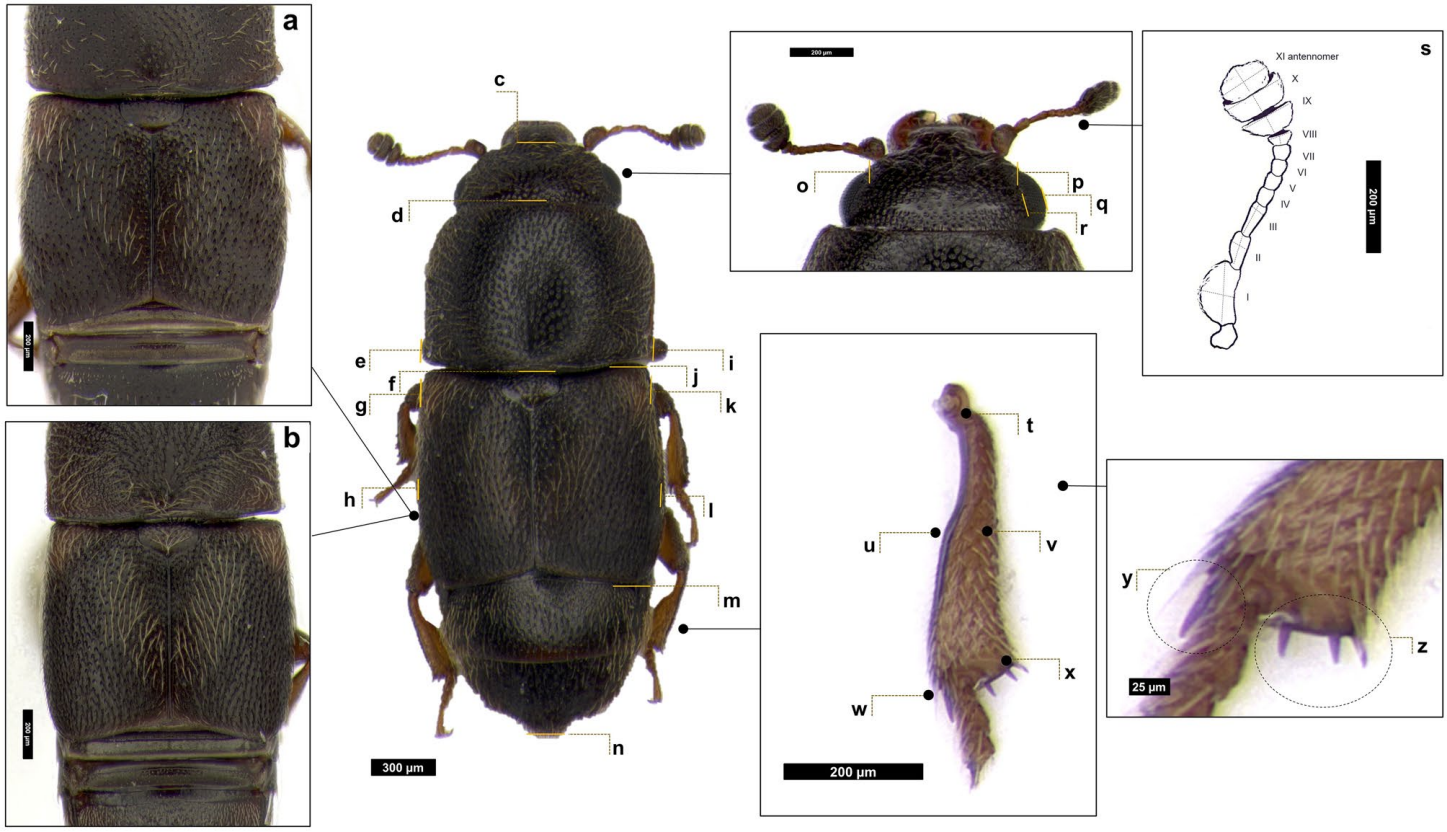
Dorsal view of *Carpophilus* *truncatus*. a-b: Coloration and apical shape of elytra, posterior shape of the pronotum. Morphometric measures: c-n: Body length; d-f: Pronotum length; j-m: Elytral length; h–l: Body width; e-i: Pronotum width; g-k: Elytral width; o-p: Intraocular distance at narrowest point; q-r: Eye width at widest point in dorsal view; s: Antennomers (I-XI) length and width; t-x: Metatibia total length; u-w: Metatibia partial length; w-x: Metatibia distal width; u-v: Metatibia proximal width; y: Number of spurs; z: Number of spines.

Sap beetle adults and larvae were examined under a Leica M165C auto montage microscope (Leica Microsystems, Mannheim, Germany) equipped with a Leica DFC450 digital photo camera to obtain multifocal images, which were then assembled with the Leica Application Suite software version 3.8.0 ^34^. Specimens were tentatively identified using the available taxonomic keys^23,32,35–39^ and original descriptions^30,40–47^. Keys and descriptions are often based only on male characters (e.g., parameres and metatibia); this makes the identification of isolated females rather difficult for species belonging to the *C. dimidiatus* complex^12,22,23,30^. Fifteen adults of each sex, randomly selected, were measured. The characters examined and how they were measured are summarized in Figs. 1﻿ and 2. For comparison, we measured also specimens of the private collection of author PA (Supplementary Table S1–S2). The following characters were also recorded: mandibles symmetry, antennomere coloration, relative length of the third and second antennomers, antennal club shape, pronotum setation length in the discal area, pronotum shape in the posterior part, tibiae’s shape, female pygidium lateral margin shape, apical margin shape, apical flexion, setation density and length.

**Figure 2:**
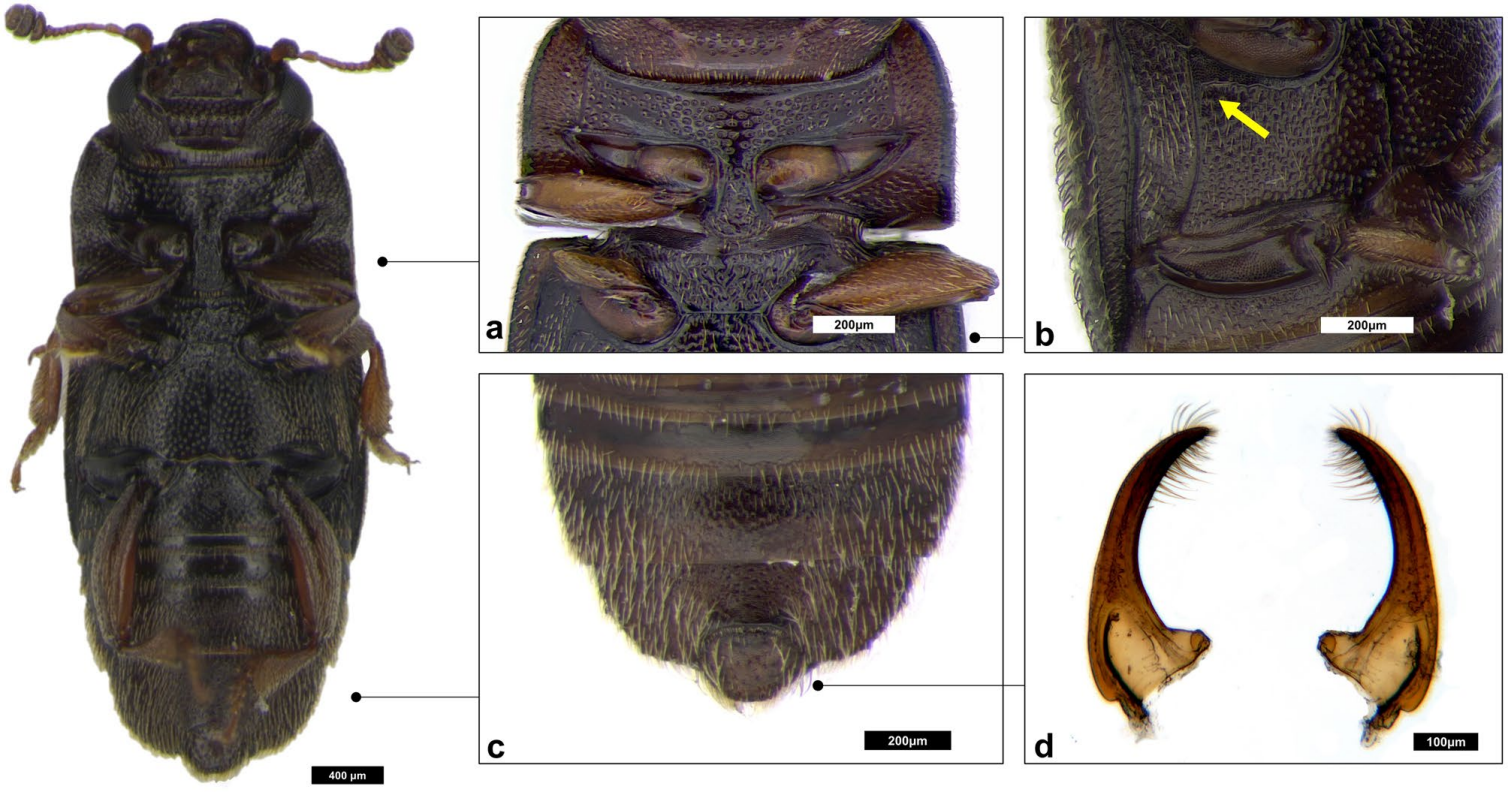
Ventral view of *Carpophilus* *truncatus*. Qualitative observation. a: absence of median longitudinal ridge on the mesosternum; b: shape of posterior rim of mesocoxal cavities and axillary space; c: setation on 5th ventrite and the supplementary segment of male and absence of depressions on 5th ventrite; d: Male genitalia, parameres (specimen AA046).

The male genitalia of specimens characterized morphologically and genetically were extracted and observed through a temporary slide with glycerin under a Zeiss Axiophot 2 microscope (Carl Zeiss, Oberkochen, Germany); they were photographed with an Axiocam HRC digital camera attached to the microscope and the combineZP® software was used to obtain multifocal images (Fig. 2d). Morphology of study specimens was then compared with that of *Carpophilus truncatus* (Murray)^28,35,44,46^ sub “*C. dimidiatus*”^47^, *Carpophilus jarijari* Powell and Hamilton^30^, *Carpophilus pilosellus* Motschulsky^23,40,45^, *Carpophilus floridanus* Fall (synonym of *C. pilosellus* sensu Gillogly)^36,41^, and *Carpophilus halli* Dobson (synonym of *C. pilosellus* sensu Connell)^22,32,37,43^.

### Molecular characterization

A Chelex and proteinase K protocol^48^ was applied to extract genomic DNA from a grinded (metathoracic) leg or from the larval head of each sample. The list of specimens analyzed is summarized in Supplementary Table S1. Molecular analysis was also performed on specimens obtained from the Audisio’s collection. Two sets of primers were used to obtain a ~ 1450 bp portion of the cytochrome c oxidase subunit I (COI): *LCO-1490*/*HCO-2198*^49^ (the barcoding region), and *C1-J-2183/TL2-N-3014*^50^. The choice of a long COI fragment derived from preliminary results showing marginal differences in Italian specimens and only in the COI barcode portion. When needed, to obtain longer and ambiguity-free sequences, primers *CO1 lco hym* and *CO1 hco outout*^51^ were used to amplify a COI fragment overlapping with the previously obtained sequences. To overcome amplification difficulties or low-quality COI sequences from museal specimens belonging to the Audisio’s collection, specific primers were designed based on COI sequences retrieved from other sequenced samples to obtain a ~ 300 bp fragment (hereafter COI-Aud). The primers *CrP-F* 5′-CCCCGGCTCACTAATCGGTA-3′; *CrP-R* 5′- ATGAACCTCCATGGGCGATA-3′ were designed using the Primer-Blast tool^52^. The ribosomal gene ITS2 along with the expansion segments D1-D2 of the 28S ribosomal subunit (28S-D1-2) (for an amplicon size of ~ 1200 bp) were amplified with primers *ITS2F* and *D2R*^53^. PCR amplifications were performed on an Eppendorf Nexus GX2 thermocycler in a 10-µl reaction volume: 5 μl DNA-free H_2_O, 2 μl 5X colorless GoTaq® reaction buffer (Promega), 0.8 μl of 0.25 µM dNTPs, 0.5 μl of each 10 µM primer, 0.2 μl of GoTaq® DNA Polymerase (Promega), and 1 μl of template DNA. Thermocycler conditions for COI fragments were set as reported in^48^ with the modification of the annealing temperatures to 50 °C and 57 °C for the pairs *C1-J-2183/TL2-N-3014* and *CrP-F/CrP-R*, respectively*.* The PCR cycling program to amplify ITS2 and 28S-D2 was 3 min at 94 °C; 35 cycles of 45 s at 94 °C, 1 min at 52 °C, and 1 min at 72 °C; with a final extension at 72 °C for 5 min. PCR products were checked on a 1.2% agarose gel stained with Xpert Green DNA Stain (20,000X) and directly sequenced. Generated sequences were deposited in GenBank under the accession numbers reported in Supplementary Table S1 .

COI sequences were aligned using the ClustalW algorithm included in BioEdit^54^, ITS2-28S sequences (hereafter nuclear regions) were aligned as a whole using the G-INS-I algorithm in MAFFT 7^55^ and this alignment was used for concatenated analysis. Ribosomal regions were also used separately in single-gene phylogenetic analyses. To perform phylogenetic reconstructions, all the homologous sequences of *Carpophilus* available in GenBank and BOLD were included in the analyses (www.ncbi.nlm.nih.gov/genbank/; www.boldsystems.org; last accessed on 21 November 2021) setting a threshold of 70% similarity with the sequences obtained.

Not all markers were available on GenBank (or BOLD for COI) databases for each species, hence different genes or their portions were analyzed both separately and combined to get a clearer picture of phylogenetic relationships. Phylogenetic reconstructions were performed by conducting a maximum likelihood (ML) analysis in RAxML version 8.2.10^56^ using the RAXMLGUI v. 2.0.5^57^ for every marker (COI, ribosomal regions and combined molecular markers, and Bayesian inference (BI) in MrBayes 3.2^58^ for the COI barcode portion and combined molecular markers. For the ML and BI analyses, the GRT + G + I evolutionary model selected by jModeltest^59^ was used for each genetic portion except ITS2, for which GRT + G model fit better. The phylogenetic reconstruction based on a concatenated dataset (COI + ribosomal regions) included all the *Carpophilus* species for which we obtained sequences of all markers for at least one specimen, and those available on public databases that were related to our samples based on single-gene phylogenetic analyses. Partitioned evolutionary models, according to the best partitioning scheme found by Partition Finder^60^, were used for the BI analysis on concatenated dataset. Trees were rooted by setting *Aethina tumida* (Coleoptera: Nitidulidae, Nitidulinae) as outgroup for COI and 28S or using the midpoint-rooted tree option for markers where outgroups were not available (acc. num.: KP134137 and KP134071). Phylogenetic trees were drawn by using FigTree software v1.4.2^61^. COI genetic distances within and between lineages were calculated using the *p*-distance method in MEGA 6, only for the COI barcoding groups our samples clustered in^62^.

### Intraspecific haplotype diversity assessment

Two methods were used to estimate the necessary sample size to evaluate the intraspecific genetic diversity of *Carpophilus*. Coalescent theory^63^ was used to calculate the probability (*p*) of sampling all haplotypes of the species with sample size (n) using the Eq. (1)^64^:

1 $$p=\frac{n-1}{n+1}$$

The Eq. (1﻿) developed by Grewe et al. ^65^ was used to calculate the minimum sample size to exclude the presence of a given haplotype in a locality:

2 $$\mathrm{n}=\frac{\mathrm{ln}(1-\beta )}{\mathrm{ln}(1-p)}$$

where *p* is the frequency of a given haplotype, and β is the desired confidence level.

### Reconstructing the most likely area of introduction through the Geographic Profiling (GP) approach

To identify the possible areas of origin for the spreading of the population of sap beetle in Campania, a Geographic Profiling (GP) approach was used^66–68^. Although several GP algorithms include the presence of a buffer area, we used the Dragnet algorithm that excludes a buffer zone, because the dispersal capabilities of small coleopterans are rather limited^67,69,70^ and the spreading of small coleopteran pests is often more dependent on human activities than dispersal capabilities of the species^71^, but see exceptions^72,73^. To gain a more robust prediction, we used a bootstrap approach^74^ by repeating the Dragnet algorithm on 30 subsamples of the whole dataset, with each subsample including a random amount of the original data ranging between 25 and 75%. Finally, the mean of the scores was computed from the different models, as this approach has been shown to render the areas of highest priority (specificity), along with the surrounding area that can be considered analogous to confidence limits^74^. Because in Campania no individuals of the sap beetle under study were found in walnut fruits above 600 m a.s.l. (Supplementary Table S1), the reconstruction for the final mean model to all the local areas (to estimate the most likely area of first introduction) was restricted from 0 to 600 m a.s.l.

### Statistic analysis

The ANOVA test was used to analyze data when the conditions of normality and homoscedasticity were respected. In the case where the conditions of normality and homoscedasticity weren’t satisfied, the non-parametric test Kruskall–Wallis was used, while a Box and Whiskers plot analysis was performed to separate medians graphically^75^.

## Results

### Monitoring activities

**Figure 3:**
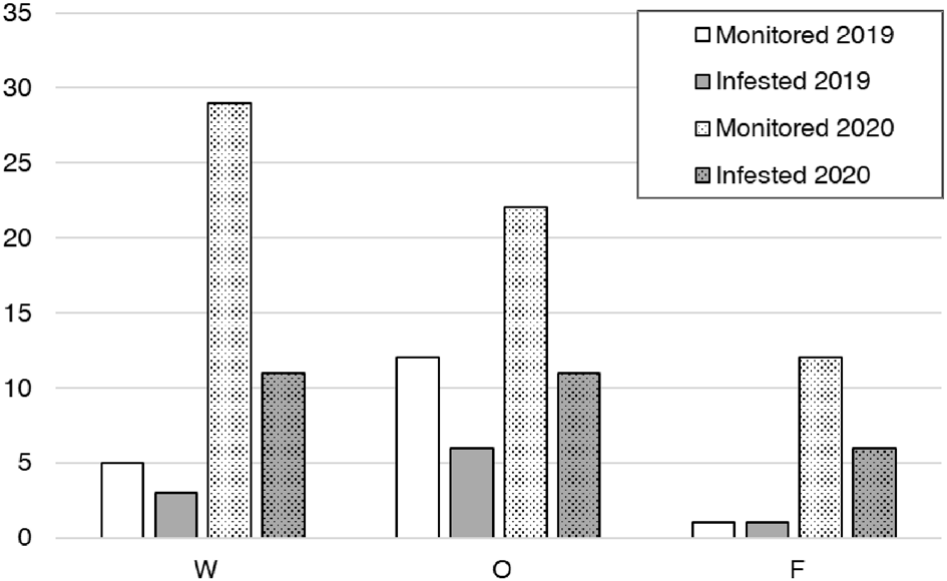
Subdivision of monitored and infested sites monitored in 2019–2020. Warehouses: W; walnut orchards: O; farms: F.

**Figure 4:**
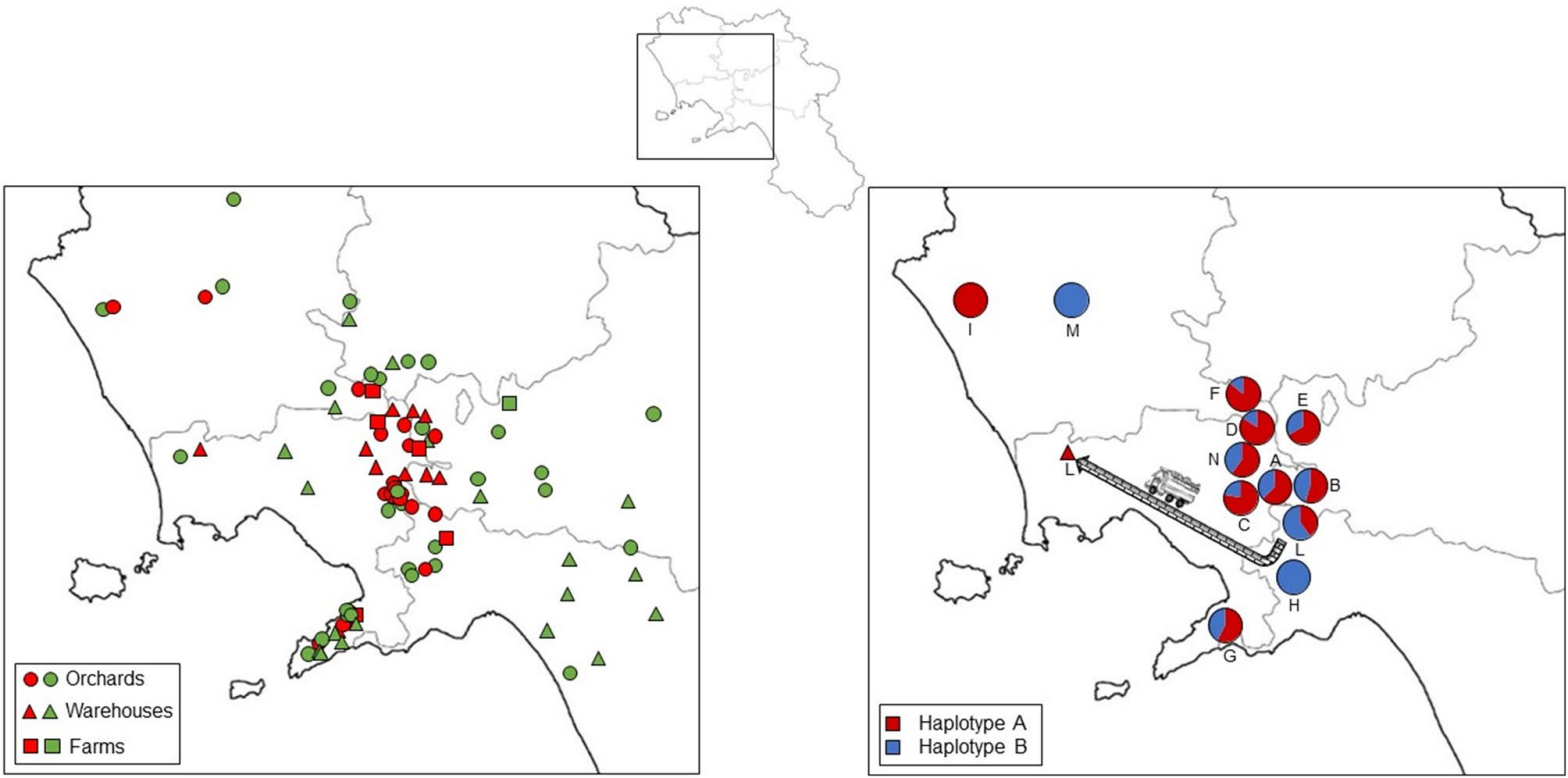
Survey area of monitoring activities in 2019–2020. On the left: position and types of sites where walnut fruits were sampled; infested sites (red) and uninfested sites (green). On the right: distribution and frequency of haplotypes; each circle represents an area of 3 km^2^ which includes multiple infested sites.

In 2019, 10 sites out of 18 (55.5%) were infested by *Carpophilus* sp. and 82 adults and 199 larvae were collected. In 2020, 28 out of 63 monitored sites (45.3%) resulted infested and 106 adults and 1123 larvae were collected (Fig. 3, 4). The northernmost plantation site, where *Carpophilus* was recorded, was located in Pignataro Maggiore (Caserta province), while the southernmost site was located in Massa Lubrense (Naples province). The plantation situated in Vico Equense (Naples province) was the one with the highest altitude (549 m a.s.l.). The walnut fruits stored in warehouses came from surrounding plantations, except for the walnut fruits collected in Sarno (Salerno province; L circle area in Fig. 4 on the right) that were moved to a warehouse in Giugliano in Campania (Naples province; L red little circle in Fig. 4 on the right). Locations A, B, D, E, and F (areas with a diameter of 3 km^2^ where *Carpophilus* was found) (Fig. 4) are usually used for cultivating of hazelnuts^76^. Locations C, I, L, M, and N were mainly situated in a flat area; some sites were near urban area, others were in an agricultural context (vegetable crops and fruit orchards). Location H and one site of the location L were located in more forested and hilly areas. Location G concerns the sites located on the Sorrento peninsula, where citrus are cultivated almost exclusively^76^.

### Damage

The main evidence of *Carpophilus* in walnut fruits was the presence of different life stages or of light frass up to 0.1 mm diameter consisting of residues of walnut and excrements (Supplementary Fig. S1). Larvae and adults of *Carpophilus* dug tunnels flattened in the cross section on the kernel, eating the kernel’s center and leaving the darker skin on the outside intact. Sometimes, both sap beetles and moths infested the same walnut (about 3% of the walnuts examined). If no live insects or parts of them were found, the damage was attributed to moths if silk threads or frass larger than 0.5 mm diameter were found. The damage caused by *Carpophilus* sp. on walnut fruits is summarized in Table 1. No significant differences were found in the average number of sap beetles present within the walnut fruits of the different sampling types in 2019 (ANOVA F_3,81_ = 2.32, *p *= 0.017) (Table 1). Conversely, in 2020, there was a significant difference in the mean number of sap beetles present within the sorted walnut fruits and the discarded ones (Kruskall–Wallis, χ^2^ = 8.94, df = 228, *p* = 0.030) (Table 1). About 80% of the fruits that resulted infested with *Carpophilus* sp. showed a crack in the sheath between the two shells of the endocarp.

**Table 1:** Percentage of fruit damaged by sap beetle and mean number of sap beetles per walnut in 2019–2020 in different walnut categories. Sorted: walnut fruits sorted by mechanical and/or manual process; unsorted: walnut fruits harvested, stored in warehouse but not subjected to a sorting process; Discarded: walnut fruits discarded during sorting process; Collected from ground: walnut fruits picked directly from the ground in walnut orchards; * denotes a statistical difference.

Walnut category	2019				2020			
	Damaged fruits		Sap beetles/walnut		Damaged fruits		Sap beetles/walnut	
	Mean ± SE	Range of variation	Mean ± SE	Range of variation	Mean ± SE	Range of variation	Mean ± SE	Range of variation
Sorted	10.0 ± 0.00	10.0 – 10.0	3.33 ± 0.667	2.0 – 4.0	5.7 ± 1.94	2.3 – 12.9	1.45 ± 0.207*	1.0 – 3.0
Not sorted	16.2 ± 6.03	5.0 – 50.0	3.96 ± 0.687	1.0 – 16.0	11.9 ± 2.74	2.0 – 27.3	3.63 ± 0.700	1.0 – 28.0
Discarded	32.5 ± 14.50	5.0 – 60.0	3.65 ± 0.688	1.0 – 15.0	24.0 ± 4.04	2.8 – 57.8	5.34 ± 0.659*	1.0 – 40.0
From ground	22.5 ± 4.23	5.0 – 35.0	2.26 ± 0.344	1.0 – 7.0	10.2 ± 2.60	1.7 – 36.7	4.08 ± 0.721	1.0 – 30.0

### Morphological characterization

Measured characters and qualitative observations are summarized in Supplementary Tables S2–S4. Results of the comparison with *Carpophilus* species that most closely resembled the species here studied are shown in Supplementary Fig. S3. The closest species were *C. truncatus* and *C. jarijari*. The main discriminating characters were very similar in these two species, namely, parameres shape (Fig. 2d) and metatibiae (Fig. 1) (abruptly restricted proximally in males): in males of *C. jarjari* abruptly dilated in their distal half/third, while in males of *C. truncatus* abruptly dilated in their distal two thirds or three fifths: Fig. 1 t-x). However, male metatibiae of all our samples resulted identical to the typical shape above recorded for *C. truncatus.*

The parameres of our samples resembled more those of *C. jarijari*^30^*,* even if remarkable similarities with parameres of *C. truncatus* description, draws, and PA’s collection samples^22,23,30,41,44,47^ were noticed. Furthermore, the shape of parameres was found to be slightly variable among samples, in fact at least one specimen out of 15 examined showed parameres smaller and not regularly restricted up to the apex. Moreover, the parameres shape is strongly influenced by the angling placement on the slides, so a slight pressure on the slide cover, modifying their inclination, has a strong influence on the photo. This character was not correlated with the haplotype. The specimens of the most frequent species found in Italy were all identified as belonging to *C. truncatus* (= *pilosellus* auct., nec Motschulsky).

### Molecular characterization

Mitochondrial COI sequencing revealed two haplotypes (Supplementary Table S1). BLAST search revealed that the haplotype B (hB) has 100% similarity with *Carpophilus “dimidiatus* (F.)” (accession number MG679359) found in Argentina ^28^; the haplotype A (hA) was recovered for the first time and differed from hB by 22/1448 bp. All the Italian *C. truncatus* samples, collected from different localities and on different dates, shared the same ribosomal genes sequence. No amplification was obtained from museal samples, except for samples AA103 and AA104, from which the COI-Aud portion was sequenced. These short sequences were identical to each other and to hB.

**Figure 5:**
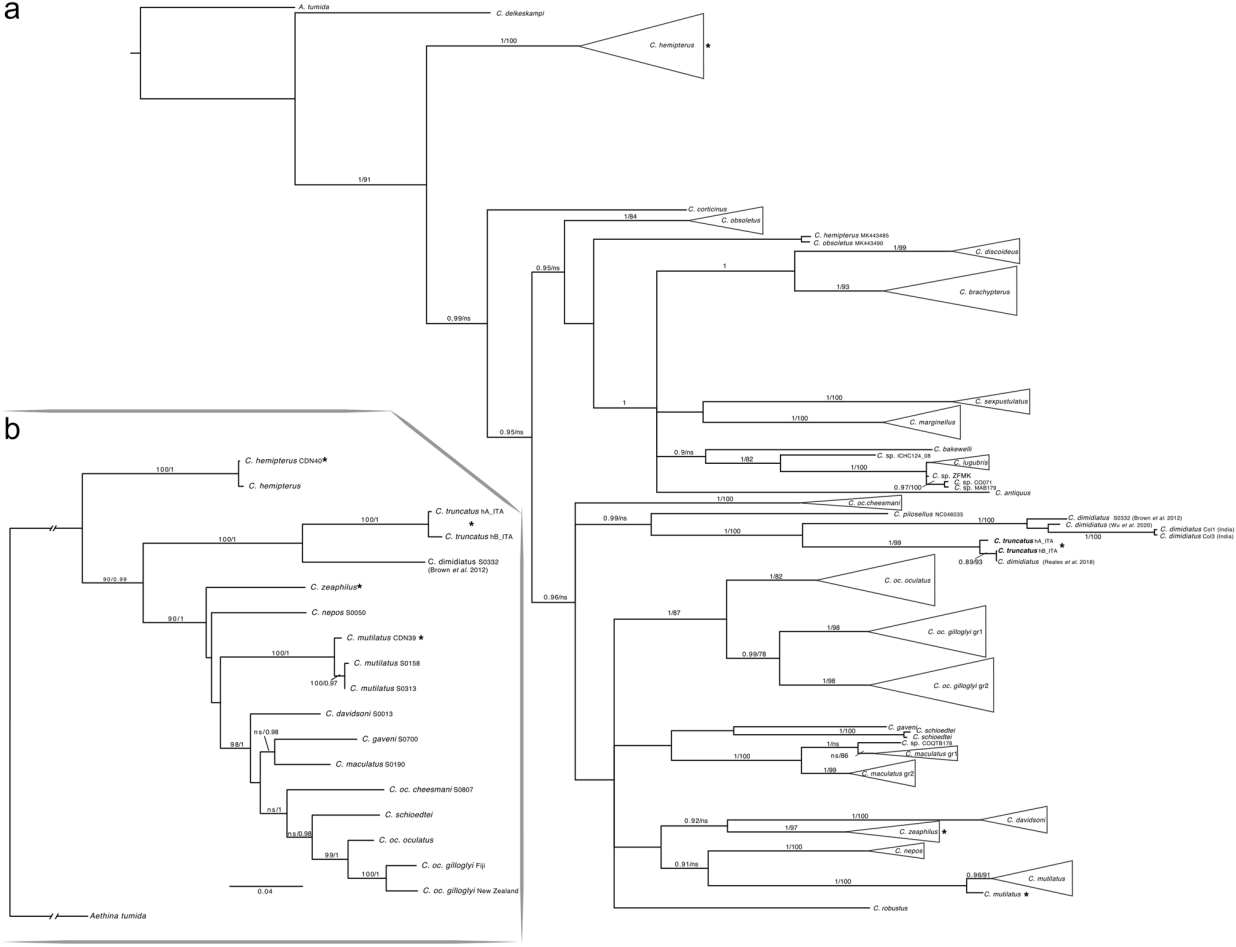
BI majority-rule consensus tree for (**a**) COI barcoding portion and (**b**) concatenated COI + ITS2 + 28S-D2 genes of *Carpophilus* spp. Posterior probability values > 0.90 are shown above branches, along with bootstrap values > 70% for the topologically identical ML tree based on 10,000 rapid pseudoreplicates. Two parallel runs of four simultaneous Monte Carlo Markov chains were run for 5 and 1 million generations for COI barcoding and concatenated alignments respectively. ns = not significant; * indicate the placement of the samples here studied.

ML and BI phylogenetic reconstructions based on the barcoding region resulted in trees of identical topology but often with different statistical support (Fig. 5a). Our *Carpophilus* samples, from the field and from the Audisio’s collection, belonged to three different groups. Both mitochondrial haplotypes of *C. truncatus* clustered with *C. “dimidiatus”* sensu Reales et al.^28^ from Argentina, and this cluster was sister to the *C. dimidiatus* group (specimens from French Polynesia, China, and India) and *C. pilosellus* (represented by a single specimen), according to the recent reinterpretations of this taxon^38,39^. The last group showed *C. davidsoni* as the sister group of *C. zeaphilus* (CM45-CM44 from *Malus* sp.; AA049 from walnut) and *C. nepos* as the sister group of *C. mutilatus*. Phylogenetic reconstructions based on ribosomal genes (Supplementary Fig. S4) (GenBank accession numbers from ON555771 to ON555778) were partially congruent with that obtained with COI (for 28S-D2: CDN40 clusters with *C. hemipterus* while *C. truncatus* is sister group of the true *C. dimidiatus*). However, as shown more clearly by ITS2 phylogenetic reconstruction, the lack of the sequences of many species did not allow a complete analysis (data not shown). Phylogeny based on combined COI + ribosomal genes (Fig. 5b) showed that the phylogenetic relationships among the samples here studied were congruent with COI reconstruction (Fig. 5a).

Uncorrected *p*-distances based on COI barcode within and between groups are reported in Table 2. The mean distance across the species was 14.8%. The highest interspecific distance was between *C. dimidiatus* and *C. davidsoni* (18.5%) while the lowest between *C. davidsoni* and *C. zeaphilus* (10.3%). The mean intraspecific distance was 1.39%, *C. dimidiatus* showed the highest value (3.52%) while the lowest value (0.62%) was found in *C. truncatus* and *C. zeaphilus*.

**Table 2:** Uncorrected *p*-distance values based on COI barcoding portion of *Carpophilus* spp. Interspecific distances (± SE) are below diagonal, intraspecific distances (± SE) are in italic along the diagonal. n.c.: not calculable.

	*C. davidsoni*	*C. dimidiatus*	*C. truncatus*	*C. hemipterus*	*C. mutilatus*	*C. nepos*	*C. pilosellus*	*C. zeaphilus*
*C. davidsoni*	*0.75* ± *0.004*							
*C. dimidiatus*	18.49 ± 0.014	*3.52* ± *0.006*						
*C. truncatus*	17.36 ± 0.012	12.86 ± 0.013	*0.62* ± *0.002*					
*C. hemipterus*	15.13 ± 0.012	17.46 ± 0.014	15.61 ± 0.012	*1.30* ± *0.002*				
*C. mutilatus*	13.52 ± 0.013	16.19 ± 0.013	15.72 ± 0.013	16.43 ± 0.013	*1.02* ± *0.0003*			
*C. nepos*	14.55 ± 0.014	16.01 ± 0.013	14.52 ± 0.014	13.99 ± 0.014	12.55 ± 0.014	*1.94* ± *0.004*		
*C. pilosellus*	15.28 ± 0.015	15.44 ± 0.013	13.36 ± 0.014	14.04 ± 0.014	14.90 ± 0.014	14.88 ± 0.012	*n.c*	
*C. zeaphilus*	10.33 ± 0.012	17.37 ± 0.014	15.00 ± 0.014	14.82 ± 0.014	12.85 ± 0.015	11.50 ± 0.015	14.72 ± 0.016	*0.62* ± *0.002*

### Intraspecific haplotype diversity assessment

Equation (1) showed that, although we examined 67 samples, 39 samples would have been sufficient to recover the genetic variations of the species (*p* = 95%). Only two mitochondrial haplotypes (A and B) were observed. Following an iterative approach, and starting from the preliminary frequency of the two recorded haplotypes, the minimum sample size resulted in 6.5. Both haplotypes were present in almost all localities (9) where at least two specimens were found. The frequency of the two haplotypes, calculated on the total number of sequenced specimens, was 53.8% for hA and 46.2% for hB (Supplementary Table S1; Fig. 4).

### Reconstructing the most likely area of introduction through the Geographic profiling (GP) approach

**Figure 6:**
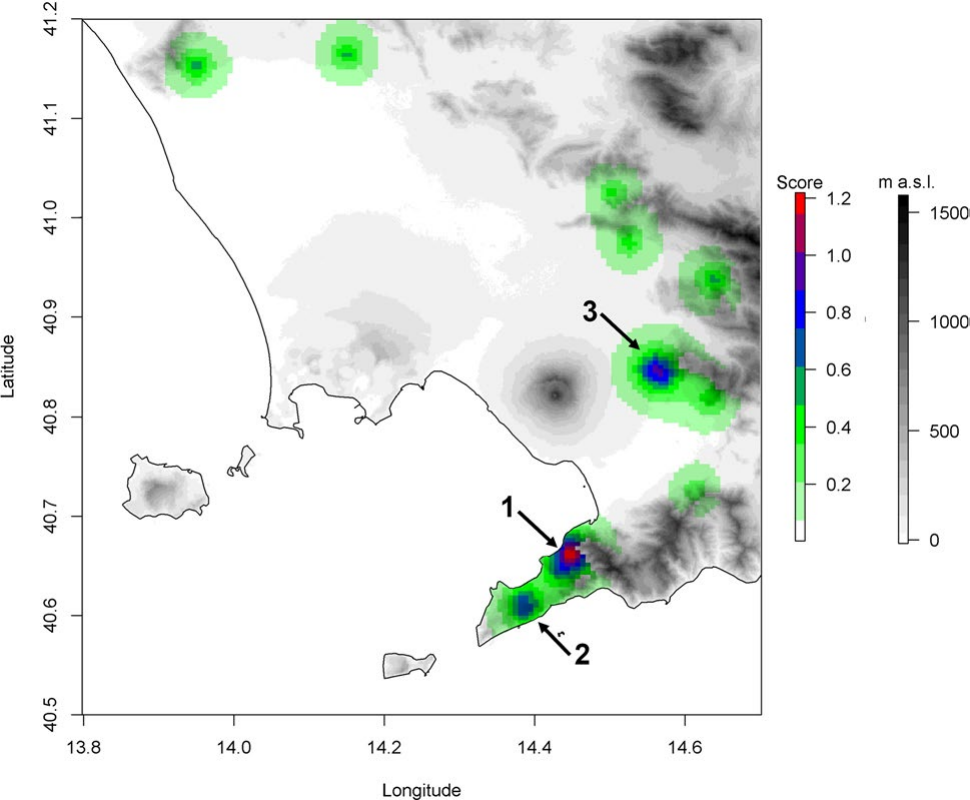
Rendering of the mean prediction for the Dragnet GP algorithm after bootstrapping. The areas are colored according to the score value of the prediction, which can be seen and increasing likelihood for the origin of the spreading for *Carpophilus truncatus* in Campania. The areas above 600 m a.s.l. have been discarded from the prediction.

The mean prediction after the bootstrapping procedure of the Dragnet GP algorithm is shown in Fig. 6. The model rendered three areas as the potential source for the spreading of the pests, two of them along the Sorrento peninsula (Fig. 6.1 and 6.2) and one between the Vesuvio and the mountains above Palma Campania (Fig. 6.3). In particular, the area with the highest score is located in the foothills of Monte Faito, one of the main peaks of the Lattari mountains, around the municipality of Vico Equense (Fig. 6.1). The latter area is the most likely area of origin for the spread of *C. truncatus* in Campania, while the other areas are secondary sources.

## Discussion

The correct identification and complete characterization of a harmful insect species is the first and indispensable step for any type of pest control. Sometimes, however, due to the scarce or absent knowledge of some emerging species and of the taxonomic groups to which they belong to, this first phase is an almost insuperable challenge^16^. Also in this case, the difficulties encountered in the identification of the species under study were considerable, due to the inadequacy of morphological and molecular knowledge of several *Carpophilus* species. However, our ultimately successful and comprehensive characterization of this species will allow for an easy identification in the future. Many species of *Carpophilus* have been described by observing a few or a single specimen, thus providing little if any data on the variability of each character. Having examined here 15 females and 15 males, we now also have a much better understanding of the intraspecific variability, including some characters considered diagnostic.

Our integrative approach has shown that the specimens found in Italy belong to *C. truncatus*. However, the identification process highlighted issues with the identification by other authors. Specimens collected in Italy share the same mitochondrial haplotype (hB) with specimens collected in Argentina on stored walnuts^28^ and with specimens collected in Australia on almonds^29^. Although Reales et al.^28^ adopted an integrated approach as well, it is evident that the species was misidentified. The authors identified the specimens collected as *C. dimidiatus* even though the morphology did not match the description, and the COI was not similar enough to other *C. dimidiatus* sequences deposited in GenBank. The identification of the Australian specimens is instead more controversial. Australian specimens were temporarily named *Carpophilus* near *dimidiatus* ^29^ and were successively described as the new species *C. jarijari*^30^, while other authors concluded that those individuals belong to *C. truncatus*^31^. *Carpophilus jarijari* is morphologically very close to *C. truncatus* and according to its description, it is distinguished by the particular shape of the parameres^30^. However, we have highlighted that the parameres exhibit a degree of variability that is not considered in any key, and the perception of their shape is strongly affected by the methodology with which this character is evaluated. This variability, together with the molecular correspondence between *C. truncatus* and Australian samples^29,77^, creates a need for the taxonomic position of *C. jarijari* to be re-evaluated. The unreliability of some morphological discriminating characters in this genus, if used alone, has been known for a long time^32^.

Molecular identification was hindered by the absence of sequences under the name of *C. truncatus* in the Genbank and BOLD databases. Furthermore, the presence of a sequence of *C. pilosellus* (in GenBank) renders the whole scenario even more complex. *Carpophilus pilosellus* Motschulsky (original description) is considered a synonym of *C. mutilatus* by^78^ and a synonym of *C. dimidiatus*^44^, but recent data^38,39^ consider the existence of the true *C. pilosellus,* as a distinct and valid species, at least in southern North America (Supplementary Table S5). Moreover, the descriptions of *C. pilosellus* after the original one refers instead to *C. truncatus*^23^ in Genbank (accessions NC046035-MN604383) entangled the identification process. However, the analysis of the interspecific *p*-distances between *C. pilosellus* and *C. truncatus* showed a genetic distance (13.4%) too much higher than the mean and the maximum intraspecific distance found in examined *Carpophilus* spp. (1.39% and 3.52% respectively) and this can have two possible explanations:

- *C. pilosellus* Motschulsky, 1858 (not *pilosellus* auct.) is truly a valid species (as informally introduced by Di Lorenzo et al.^38,39^) and the synonymy with *C. dimidiatus* or with *C. mutilatus* should be withdrawn;
- The sample to which the sequence belongs was misidentified and it belongs to an undescribed species or is to be referred to another taxon within the complex.

Phylogenetic reconstruction based on different genes highlighted several apparently polyphyletic “species” (Fig. 5a,b – Supplementary Fig. S4, S5), which confirms the existence of huge identification problems inside the genus *Carpophilus*. *C. truncatus* consistently resulted sister to *C. dimidiatus*, who in turn resulted polyphyletic based on 28S and the 3’ region of COI (Supplementary Fig. S4), where the samples identified by Cline in^79^ were included in one clade. Therefore, it is imperative to try using a different strategy. On the basis of both current results and results on other taxa^80–83^, the approach based on the combined molecular markers (COI + ITS2 + 28S-D2) is quite effective and should be used for future studies of this group.

The specimens under study also share the same mt haplotype (hB) and are morphologically identical to two specimens of *C. truncatus* from the Audisio’s collection. This result confirms that this haplotype was already present at least since 1996 in Italy (Supplementary Table S1), where until a few years ago it was sporadic and not harmful^23^. Some data on the species distribution and population density are unknown or not exhaustive, but the species is reported to be present but rare, in Italy and Europe since the 1800s and it never caused concern for agricultural production until 2019 (Supplementary Table S6). However, starting from 2013 in Australia^29^ and Argentina^28^ sub “*C. dimidiatus*”, and since 2019 in Italy^21^ sub “*dimidiatus*”, *C. truncatus* has begun to be harmful. The almost simultaneous appearance of a harmful population of *C. truncatus* in three different continents lets to consider it as an invasive species of stage V, widespread and dominant, sensu Colautti and MacIsaac^84^. What happened? A change has clearly occurred in the relationship between *C. truncatus* and its current hosts/habitat in recent years, and some hypotheses may be formulated to explain this process:

1. The delay in the identification of an invasive species is, unfortunately, a recurring problem; often some species are not considered potentially invasive because they do not cause damage in the country of origin^85^. It is common for an invasion to be characterized by initial periods of inactivity that suddenly evolve into an explosion^85^. This period is called *lag time* and it is referred to the invader’s impact. However, if we consider that *C. truncatus* was already present on the Italian territory since the 1800s, that this species never impacted agricultural production until 2019^23,86^, and that instead there was the almost simultaneous appearance of harmful populations in three different continents, this hypothesis becomes unlikely.
2. The explosion of the species, which consists of an increase in population density and harmfulness, could have been caused by the recent invasion of other insect species such as *R. completa* or *Apomyelois ceratoniae* (Zeller) (Lepidoptera: Pyralidae), which might create more favorable conditions for *C. truncatus* damage^20,87^. These insects indeed damage the hull and uncover the fruits, favoring an early infestation by *C. truncatus*. A similar behavior was also observed in Australia, where *C. truncatus* infests almond fruits only when the nuts are uncovered^29^. Other *Carpophilus* species, such as *C. hemipterus*, have similar opportunistic habits, penetrating peaches through damage caused by other pests^27,88^.
3. Some changes in the practices related to the management of walnut orchards and fruits (pest control, sorting process, harvest) may have influenced the explosion of this population. Until a few years ago, broad-spectrum insecticides were used in Italy and in other parts of the world to control many walnut pests^89^. Recently, many active ingredients, like dimethoate, were banned^90^. The pesticides currently used, not being systemic, may not be effective against *C. truncatus*.
4. An alternative and more probable hypothesis is that a more virulent population has only recently developed that has also been introduced in Italy. This hypothesis is also corroborated by the evidence that the Italian population has adapted to a climate zone very different from that of origin and from the one where it has been harmful until now (see below). The genetic variability within the Italian population is very low, with only two haplotypes recorded thus far. This is a recurring pattern in invasive species and has been found multiple times^6,13,91^. To date, only one haplotype has been found in Argentina and Australia, but with a negligible number of specimens analyzed^28,29^. Recently, the sharp contraction of local production of walnuts, which are being replaced by the more profitable hazelnuts, has led to an increase import of walnuts from Argentina and Australia (bridgehead scenario) and from other unidentified areas (walnut growers, personal communication). This import may have introduced a more virulent population of *C. truncatus* than the one present so far. A similar phenomenon was recently recorded in Taiwan where the introduction of a non-native lineage resulted linked to a recent outbreak of the ant *Dolichoderus thoracicus* (Smith)^92^. The use of other markers, such as microsatellites or RAD-Seq, could confirm this hypothesis.

It is also likely that two or more scenarios listed above have contributed synergistically to favor the explosion of *C. truncatus* populations. The extent of the infestation and damage caused by the sap beetle in Italy is further evidence that the complex of walnut pests is constantly and rapidly changing, as recently reported^20^. Moreover, field sampling and observations have shown that *C. truncatus* adults lay their eggs already on the fruit on the trees. Therefore, the harvested fruits are already infested and this observation is congruent with the almond fruits observed in Australia^29^ and with the evidence that at least 20% of the infested ripe nuts did not present any alteration of the sheath that closes the two fruit valves. The damage caused by *C. truncatus* larvae and adults is relevant (up to average values of 22% on fruits collected on the ground) and is both direct and indirect. The direct damage is due to the feeding activity on the kernel, while the indirect one is due to the symbiotic fungi and bacteria carried by insects, and the difficulty in the detection of the infested fruits by external examination of the walnuts. There are no shell aesthetic deteriorations and, if the damage is not advanced, there are no significant weight variations of the fruits; therefore, the infested fruits can pass the sorting process, bringing inside the warehouses specimens that can thrive in such conditions. The sorting systems used to date are ineffective at discarding infested walnut fruits. The undetectability is particularly detrimental to walnut fruits, which are still widely commercialized with shells, unlike almonds. *Carpophilus truncatus* can overwinter inside the walnut fruits in the warehouse but also in the mummified fruits in the fields. Field and warehouse sanitation could therefore positively affect the state of infestation^29^. Although the type of *C. truncatus* was collected in Madagascar^35^, the current distribution of *C. truncatus*, suggests that this species could expand its diffusion where there are the world's largest producers of walnuts and almonds.

The intense monitoring performed in the Campania region allowed to assess that the distribution of sap beetles is patchy, with several areas still unaffected. Transporting of walnut fruits from the orchards to a distant warehouse seems to have played an important role in the dispersal of *C. truncatus*. An example of this phenomenon has been recorded in the case of walnut fruits collected in Sarno (SA) and transferred to Giugliano in Campania (NA) to be sorted (Fig. 4). However, there are two areas in the Sorrento Peninsula (Fig. 6.1, 6.2) and one in the area of Palma Campania (Fig. 6.3) where the presence of the sap beetle is more frequent. The orography of the territory, influencing the climatic conditions and the habitat quality, has certainly influenced the spread of the sap beetle, but the rather uneven distribution of walnut orchards in Campania also played an important role obviously. In Campania, few specialized walnut orchards are present, but walnut is a widespread tree and it is cultivated in many private gardens. However, the prediction made with the Dragnet GP algorithm indicates that the Sorrento peninsula (Fig. 6﻿.1) is the most likely area where the pest has begun to spread. This result also seems to confirm that the recent demographic and harmful explosion of *C. truncatus* started from the recent import of walnuts, which has introduced an invasive population. It is important to remember that walnuts of the Sorrento *cultivar* are very renowned and that the contraction of local production has led to the importation of walnut fruits mainly from South America (pers. obs.). Some surveys were also carried out in other Italian regions (in Veneto, Lazio, and Calabria regions) but no specimens of *C. truncatus* were collected (Carmelo Bonsignore, Raffaele Sasso, Luca Mazzon, personal communications). This result is congruent with what is stated in previous studies that define *C. truncatus* as a *Carpophilus* species that probably encountered greater difficulties in acclimating in Euro-Mediterranean areas, where its presence is still relatively sporadic and mostly limited to urban and suburban areas or areas with anthropogenic influence^23^. At the moment the biological data about *C. truncatus* and in particular on this invasive population that infests walnuts, are scarce, but several other carpophiline species (such as *Carpophilus hemipterus*, *C. nepos* Murray, *C. dimidiatus, Urophorus humeralis* F.), due to their tropical origin, have a remarkable tolerance to high temperatures^93–95^. In Italy, *C. dimidiatus*, *C. nepos*, *C. hemipterus*, *C. mutilatus*, and *C. quadrisignatus* Erichson complete about 5–6 generations per year, with a peak of adults in summer and late summer^88^. *Carpophilus truncatus* seems instead to be univoltine in the field but it is able to complete more generations in the warehouse. The absence of *C. truncatus* in higher altitude areas (> than 250 m a.s.l.), except some findings in the Sorrentine peninsula (300–550 m a.s.l.), where the sea strongly mitigates the temperatures, seems to indicate that it has similar thermal needs of other species of the same genus (Figs. 4, 6). These results match with the observation that several *Carpophilus* species are more harmful at low altitudes^96^.

## Conclusion

*C. truncatus* has been characterized by integrating multiple data, hence making its identification much easier from now on. The same population and, in particular, one of the two haplotypes found in Italy is behaving as an invasive species on three different continents. The multilocus phylogenetic reconstruction showed that several species are apparently polyphyletic, highlighting major taxonomic problems within the genus. The infestation occurs when the walnuts are still on trees, and on average 22% of the fruits are infested. The invasive process in Italy has started from the Sorrento peninsula. Lastly, *C. truncatus* appears capable of acclimatizing to the main walnut and almond production areas of the world. *C. truncatus* has all the features to be considered a quarantine pest.

## Supplementary Information

Supplementary Information 1.

Supplementary Information 2.

Supplementary Information 3.

Supplementary Information 4.

Supplementary Information 5.

Supplementary Information 6.

Supplementary Information 7.

## Data Availability

Data will be available upon the publication of the manuscript as supplementary material, furthermore all the voucher specimens are deposited in the Insect Collection of the Institute for Sustainable Plant Protection—National Research Council (IPSP-CNR), P.le E. Fermi, 1—80,055 Portici (NA) – Italy, and are available on request to Dr. Umberto Bernardo, umberto.bernardo@ipsp.cnr.it.
