# Supplementary material for: The spread of Carpophilus truncatus is on the razor's edge between an outbreak and a pest invasion
Source: Sci Rep. 2022 Nov 7;12:18841. doi: 10.1038/s41598-022-23520-2 (PMC9640586; doi:10.1038/s41598-022-23520-2)
Supplement: Supplementary file 1 — Supplementary Information 1. [file 41598_2022_23520_MOESM1_ESM.docx]

**Scientific Reports**

**Is *Carpophilus truncatus* invading the world? On the razor's edge between an outbreak and a pest invasion in progress**

Flavia de Benedetta, Simona Gargiulo, Fortuna Miele, Laura Figlioli, Michele Innangi, Paolo Audisio, Francesco Nugnes, Umberto Bernardo

*Address correspondence to

Umberto Bernardo: umberto.bernardo@ipsp.cnr.it

National Research Council, (Institute for Sustainable Plant Protection - IPSP-CNR),

P.le E. Fermi, 1

80055 Portici (NA)

Italy


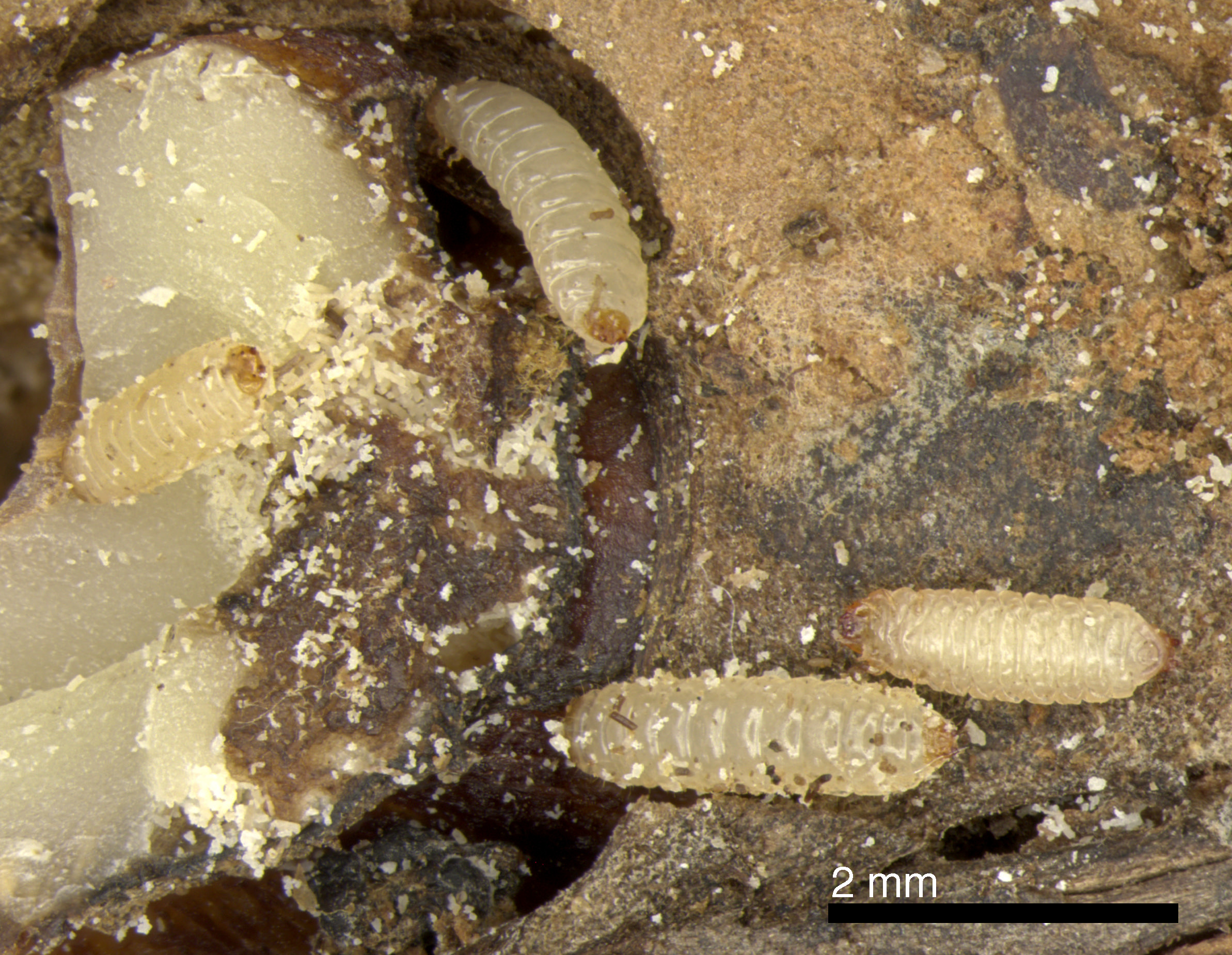


**Fig. S1** Damage caused by sap beetle larvae on walnut fruit


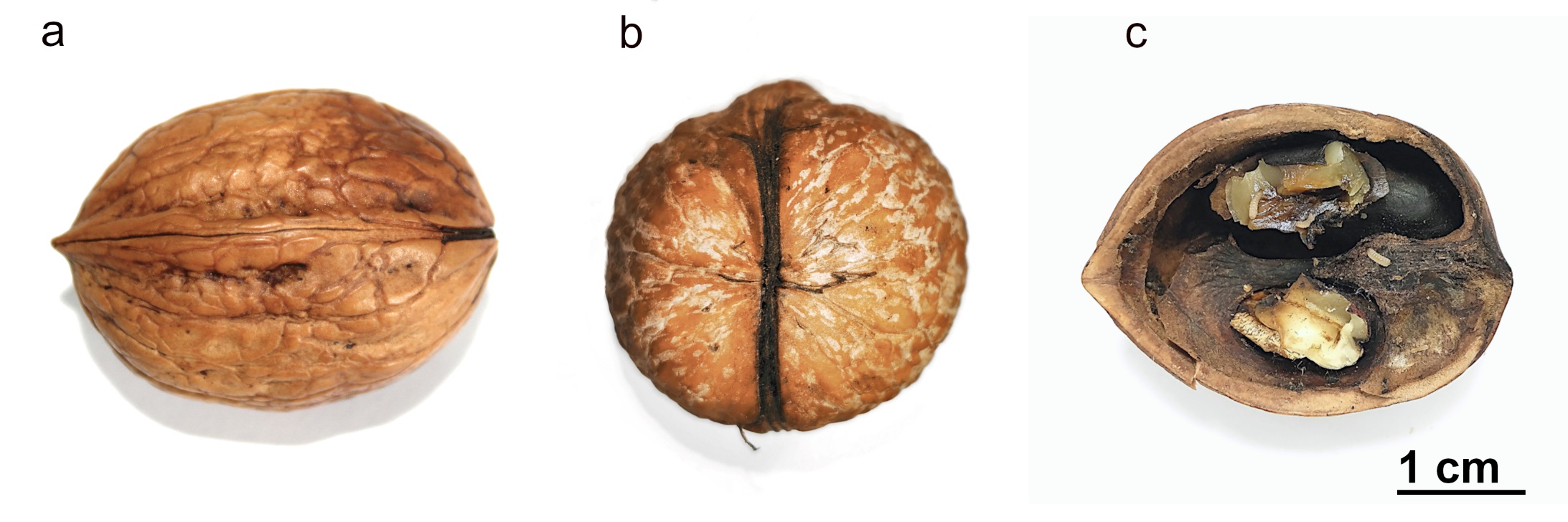


**Fig. S2** A damaged walnut. a) - b) longitudinal and orthogonal view, of apparently undamaged fruit; c) Walnut with larvae, completely damaged and unmarketable.


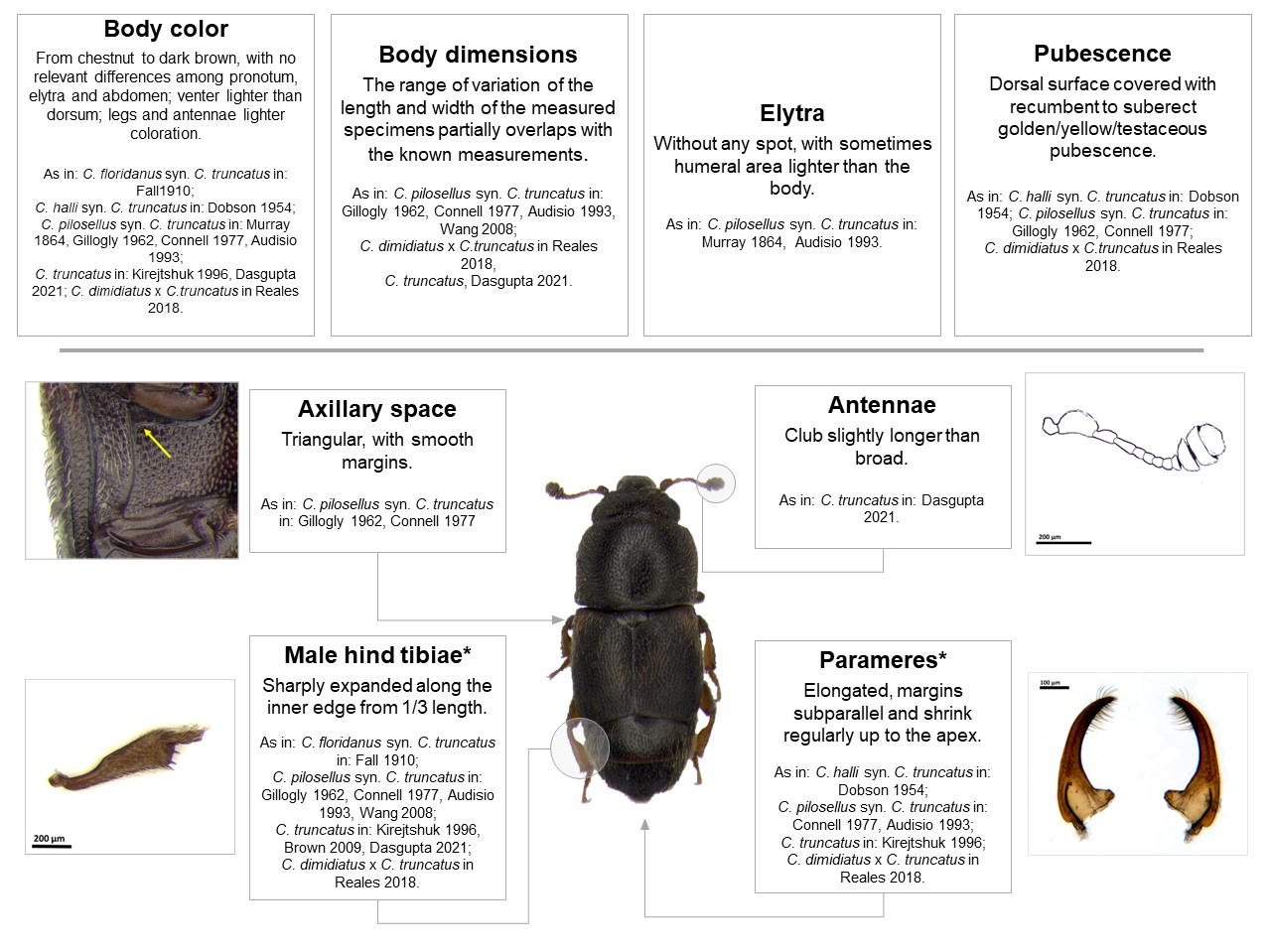
**Fig. S3** Eight important characteristics useful to correctly identify *C. truncatus*. Upper part: general aspect of body and pubescence. Lower part: small details where * indicate the species-specific, distinctive characters

**Fig. S4** ML consensus tree based on 10,000 rapid pseudoreplicates for the nuclear portion 28S-D2 of the *Carpophilus* species. Bootstrap values of >70% are shown above branches

**Fig. S5** ML consensus tree based on 10,000 rapid pseudoreplicates for the mitochondrial portion C1-J-2183/TL2-N-3014 of the Carpophilus species. Bootstrap values of >70% are shown above branches
