# Supplementary material for: The spread of Carpophilus truncatus is on the razor's edge between an outbreak and a pest invasion
Source: Sci Rep. 2022 Nov 7;12:18841. doi: 10.1038/s41598-022-23520-2 (PMC9640586; doi:10.1038/s41598-022-23520-2)
Supplement: Supplementary file 2 — Supplementary Information 2. [file 41598_2022_23520_MOESM2_ESM.docx]

| **Code** | **Date of Record** | **Stage** | **Sex** | **Host** | **Analysis** | **Species** | **MT**  **haplotype** | **City** | **Locality (3 km area)** | **Coordinates** | **Elevation** | **COI GenBank Accession code** |
| --- | --- | --- | --- | --- | --- | --- | --- | --- | --- | --- | --- | --- |
| CDA5 | 07/11/2019 | Adult | ♀ | *Juglans regia* | Mol | *Carpophilus truncatus* | hA | Carbonara di Nola | A | 40°52' N; 14°34' E | 120 | ON548244 |
| EM1 | 07/11/2019 | Larva | - |  | Mol |  | hB |  | A |  |  | ON548269 |
| EM2 | 07/11/2019 | Larva | - |  | Mol |  | hB |  | A |  |  | ON548270 |
| EM3 | 07/11/2019 | Larva | - |  | Mol |  | hA |  | A |  |  | ON548245 |
| AA074 | 30/10/2020 | Adult | ♀ |  | Morp |  | - |  | A |  |  | - |
| CDN14 | 30/10/2020 | Adult | ♀ |  | Morp/Mol |  | hA |  | A |  |  | ON548252 |
| CDN15 | 30/10/2020 | Adult | ♀ |  | Morp/Mol |  | hB |  | A |  |  | ON548277 |
| AA033 | 30/10/2020 | Adult | ♀ |  | Mol |  | hA |  | A |  |  | ON548300 |
| CDN18 | 30/10/2020 | Adult | ♀ |  | Morp/Mol |  | hA |  | A |  |  | ON548253 |
| CDB3 | 19/11/2019 | Adult | ♀ |  | Mol |  | hB | Lauro | B | 40°52'N; 14°36' E | 183 | ON548271 |
| AA071 | 30/10/2020 | Adult | ♀ |  | Morp |  | - |  | B |  |  | - |
| CDN2 | 30/10/2020 | Adult | ♀ |  | Mol |  | hB |  | B |  |  | ON548272 |
| CDN3 | 30/10/2020 | Adult | ♀ |  | Morp/Mol |  | hA |  | B |  |  | ON548246 |
| CDN4* | 30/10/2020 | Adult | ♂ |  | Morp/Mol |  | hB |  | B |  |  | ON548273 |
| CDN5 | 30/10/2020 | Adult | ♀ |  | Morp/Mol |  | hA |  | B |  |  | ON548247 |
| CDN6 | 30/10/2020 | Adult | ♀ |  | Morp/Mol |  | hB |  | B |  |  | ON548274 |
| AA072 | 30/10/2020 | Adult | ♀ |  | Morp |  | - |  | B | 40°52'N; 14°38' E | 212 | - |
| AA073 | 30/10/2020 | Adult | ♀ |  | Morp |  | - |  | B |  |  | - |
| CDN10 | 30/10/2020 | Adult | ♂ |  | Morp/Mol |  | hA |  | B |  |  | ON548250 |
| CDN11 | 30/10/2020 | Larva | - |  | Mol |  | hA |  | B |  |  | ON548251 |
| CDN12 | 30/10/2020 | Adult | ♂ |  | Mol |  | hA |  | B |  |  | ON548301 |
| CDN13 | 30/10/2020 | Adult | ♀ |  | Mol |  | hB |  | B |  |  | ON548276 |
| CDN8 | 30/10/2020 | Adult | ♀ |  | Mol |  | hA |  | B |  |  | ON548248 |
| CM43 | 13/12/2019 | Larva | - |  | Mol |  | hB | Palma Campania | C | 40°51' N; 14°33' E | 81 | ON548285 |
| AA039* | 03/11/2020 | Adult | ♂ |  | Morp/Mol |  | hA |  | C |  |  | ON548263 |
| AA040 | 03/11/2020 | Adult | ♂ |  | Mol |  | hA |  | C |  |  | ON548302 |
| AA041 | 03/11/2020 | Adult | ♂ |  | Morp/Mol |  | hA |  | C |  |  | ON548303 |
| AA042 | 03/11/2020 | Adult | ♂ |  | Morp/Mol |  | hA |  | C |  |  | ON548304 |
| AA043* | 03/11/2020 | Adult | ♂ |  | Mol |  | hA |  | C |  |  | ON548305 |
| CDC3 | 26/11/2019 | Adult | ♀ | *Juglans regia* | Mol | *Carpophilus truncatus* | hA | San Gennaro Vesuviano | C | 40°51' N; 14°31' E | 56 | ON548267 |
| CDN7 | 30/10/2020 | Larva | - |  | Mol |  | hB | Sarno | C | 40°50'N; 14°34' E | 49 | ON548275 |
| CDN9 | 30/10/2020 | Larva | - |  | Mol |  | hA |  |  |  |  | ON548249 |
| AA044 | 12/11/2020 | Adult | ♂ |  | Morp/Mol |  | hA | Cicciano | D | 40°58' N; 40°31' E | 54 | ON548306 |
| AA045 | 12/11/2020 | Adult | ♂ |  | Morp/Mol |  | hA |  |  |  | 74 | ON548307 |
| AA046 | 12/11/2020 | Adult | ♂ |  | Mol |  | hA |  |  |  |  | ON548294 |
| AA047 | 12/11/2020 | Adult | ♀ |  | Morp/Mol |  | hA |  |  |  |  | ON548264 |
| AA048 | 12/11/2020 | Adult | ♀ |  | Morp/Mol |  | hA |  |  |  |  | ON548265 |
| CM46 | 3/1/2020 | Adult | ♂ |  | Mol |  | hB | Cimitile | D | 40°56' N; 14°30' E | 36 | ON548286 |
| CDN31 | 06/11/2020 | Larva | - |  | Mol |  | hB | Avella | E | 40°57' N; 14°35' E | 197 | ON548281 |
| CM47 | 3/1/2020 | Adult | ♂ |  | Mol |  | hA | Avella loc. Santa | E | 40°56' N; 14°35' E | 167 | ON548268 |
| CM48 | 3/1/2020 | Adult | ♂ |  | Mol |  | hB |  |  |  |  | ON548287 |
| CDN33 | 06/11/2020 | Adult | ♀ |  | Morp/Mol |  | hA |  |  |  |  | ON548259 |
| CDN34 | 06/11/2020 | Larva | - |  | Mol |  | hA |  |  |  |  | ON548260 |
| CDN36 | 09/11/2020 | Larva | - |  | Mol |  | hA | Mugnano del Cardinale | E | 40°57' N; 14°36'E | 245 | ON548261 |
| AA034 | 19/11/2020 | Adult | ♂ |  | Morp/Mol |  | hA | Arienzo | F | 41°1' N; 14°30' E | 121 | ON548308 |
| AA035 | 19/11/2020 | Adult | ♂ |  | Morp/Mol |  | hA |  |  |  |  | ON548309 |
| AA036 | 19/11/2020 | Adult | ♂ |  | Mol |  | hA |  |  |  |  | ON548310 |
| AA037 | 19/11/2020 | Adult | ♂ |  | Morp/Mol |  | hA |  |  |  |  | ON548311 |
| AA075 | 19/11/2020 | Adult | ♂ |  | Mol |  | hA |  |  |  |  | ON548297 |
| AA076 | 19/11/2020 | Adult | ♂ |  | Mol |  | hB |  |  |  |  | ON548299 |
| CDN19 | 19/11/2020 | Adult | ♀ |  | Morp/Mol |  | hA |  |  |  |  | ON548254 |
| CDN21 | 06/11/2020 | Larva | - |  | Mol |  | hB | Vico Equense loc. Belvedere | G | 40°39' N; 14°27' E | 549 | ON548278 |
| CDN22 | 06/11/2020 | Larva | - |  | Mol |  | hB | Vico Equense loc. Massaquano |  | 40°40' N; 14°26' E | 358 | ON548279 |
| CDN23 | 06/11/2020 | Larva | - |  | Mol |  | hA |  |  |  |  | ON548256 |
| CDN20 | 17/11/2020 | Larva | - |  | Mol |  | hA | Vico Equense loc. Pacognano |  | 40°38' N; 14°25' E | 205 | ON548255 |
| CDN26 | 05/11/2020 | Larva | - |  | Mol |  | hA | Vico Equense loc. Sant'Andrea |  | 40°39' N; 14°26' E | 293 | ON548257 |
| CDN27 | 05/11/2020 | Larva | - |  | Mol |  | hA |  |  |  |  | ON548258 |
| CDN28 | 05/11/2020 | Larva | - |  | Mol |  | hB |  |  |  |  | ON548280 |
| CDN37 | 17/11/2020 | Larva | - | *Juglans regia* | Mol | *Carpophilus truncatus* | hB | Sant'Egidio Monte Albino | H | 40°43' N; 14°36' E | 204 | ON548282 |
| AA038* | 19/11/2020 | Adult | ♂ |  | Morp/Mol |  | hA | Falciano del Massico | I | 41°9' N; 13°56' E | 42 | ON548262 |
| AA053* | 03/11/2020 | Adult | ♂ |  | Morp/Mol |  | hB | Sarno | L | 40°49' N; 14°37' E | 282 | ON548283 |
| AA054 | 03/11/2020 | Larva | - |  | Mol |  | hB |  |  |  |  | ON548314 |
| AA055 | 13/11/2020 | Adult | ♂ |  | Morp/Mol |  | hB |  |  |  |  | ON548315 |
| AA056 | 13/11/2020 | Adult | ♀ |  | Morp/Mol |  | hA |  |  |  |  | ON548312 |
| AA069 | 13/11/2020 | Adult | ♀ |  | Mol |  | hA |  |  |  |  | ON548296 |
| AA057 | 04/11/2020 | Larva | - |  | Mol |  | hB | Pignataro Maggiore | M | 41°9' N; 14°8' E | 41 | ON548284 |
| AA058 | 12/11/2020 | Adult | ♂ |  | Morp/Mol |  | hA | San Vitaliano | N | 40°55' N; 14°29' E | 33 | ON548266 |
| AA059 | 12/11/2020 | Adult | ♂ |  | Morp/Mol |  | hA |  |  |  |  | ON548313 |
| AA060 | 12/11/2020 | Adult | ♀ |  | Mol |  | hA |  |  |  |  | ON548295 |
| AA061 | 12/11/2020 | Larva | - |  | Mol |  | hB | Saviano |  | 40°53' N; 14°30' E | 41 | ON548316 |
| AA070 | 12/11/2020 | Larva | - |  | Mol |  | hB |  |  |  |  | ON548298 |
| AA103 | 10/1996 | Adult | ♂ |  | Mol |  | hB | Genova | - | 44°24' N; 8°56' E | - | ON548317 |
| AA104 | 10/1996 | Adult | ♀ |  | Mol |  | hB |  |  |  |  | ON548318 |
| CDN40* | 03/11/2020 | Adult | ♂ | *Juglans regia* | Mol | *Carpophilus hemipterus* | - | Sarno | - | 40°49' N; 14°37' E | 282 | ON548288 |
| CDN39* | 13/11/2020 | Adult | ♂ |  | Mol | *Carpophilus mutilatus* | - | Giugliano in Campania | - | 40°54' N; 14°5' E | 57 | ON548289 |
| CDN45 | 19/11/2020 | Adult | ♂ |  | Mol |  | - | Sorrento | - | 40°37' N; 14°22' E | 123 | ON548290 |
| CM44 | 20/08/2020 | Adult | ♂ | *Malus* sp. | Mol | *Carpophilus zeaphilus* | - | Benevento | - | 41°7' N; 14°46' E | 158 | ON548291 |
| CM45* | 21/08/2020 | Adult | ♂ |  | Mol |  | - | Pignataro Maggiore | - | 41°9' N; 14°8' E | 41 | ON548292 |
| AA049 | 21/12/2020 | Larva | - | *Juglans regia* | Mol |  | - | Altavilla Irpina | - | 41°0' N; 14°47' E | 361 | ON548293 |

Table S1 Specimens of *Carpophilus* spp. used for this study. Mol: molecular; Morp: morphometric; -: not available/not applicable; *: nuclear genes sequenced
