# Supplementary material for: The spread of Carpophilus truncatus is on the razor's edge between an outbreak and a pest invasion
Source: Sci Rep. 2022 Nov 7;12:18841. doi: 10.1038/s41598-022-23520-2 (PMC9640586; doi:10.1038/s41598-022-23520-2)
Supplement: Supplementary file 3 — Supplementary Information 3. [file 41598_2022_23520_MOESM3_ESM.docx]

| **Characters** | **Males (n = 15)** | | **Females (n = 15)** | |
| --- | --- | --- | --- | --- |
|  | **Mean ± SE** | **Range of variation** | **Mean ± SE** | **Range of variation** |
| Body-L (mm) | 3.17 ± 0.086 | 2.65­3.90 | 2.99 ± 0.078 | 2.41­3.51 |
| Body-W (mm) | 1.37 ± 0.036 | 1.12­1.68 | 1.29 ± 0.034 | 1.00 ­1.50 |
| P-L (mm) | 0.94 ± 0.022 | 0.71­1.00 | 0.81 ± 0.021 | 0.65­0.93 |
| P-W (mm) | 1.29 ± 0.028 | 1.06­1.43 | 1.13 ± 0.029 | 0.93­1.28 |
| E-L (mm) | 1.18 ± 0.024 | 0.96­1.28 | 1.08 ± 0.028 | 0.84­1.25 |
| E-W (mm) | 1.20 ± 0.041 | 0.78­1.40 | 1.15 ± 0.027 | 0.93­1.28 |
| Io | 758.10 ± 20.754 | 588.00­850.50 | 606.90 ± 14.206 | 493.50­672.00 |
| Eye-W | 67.20 ± 1.554 | 57.75­73.50 | 62.650 ± 1.880 | 52.50­73.50 |
| A1-L | 149.10 ± 4.490 | 126.00­168.00 | 120.40 ± 3.530 | 105.00­147.00 |
| A1-W | 91.35 ± 2.701 | 73.50­105.00 | 79.45 ± 2.656 | 63.00­94.50 |
| A2-L | 87.85 ± 2.734 | 73.50­105.00 | 74.903 ± 1.658 | 63.00­84.00 |
| A2-W | 49.00 ± 1.220 | 42.00­52.50 | 42.00 ± 1.025 | 31.50­52.50 |
| A3-L | 93.45 ± 2.622 | 73.50­110.25 | 78.40 ±1.949 | 63.00­84.00 |
| A3-W | 39.90 ± 0.999 | 31.50­42.00 | 31.85 ± 0.350 | 31.50­36.75 |
| A4-L | 41.65 ± 1.303 | 31.50­52.50 | 32.55 ± 0.917 | 26.25­42.00 |
| A4-W | 38.50 ± 1.107 | 31.50­42.00 | 32.55 ± 0.561 | 31.50­36.75 |
| A5-L | 40.25 ± 1.323 | 31.50­47.25 | 33.25 ± 1.220 | 26.25­42.00 |
| A5-W | 39.20 ± 1.130 | 31.50­42.00 | 32.55 ± 0.917 | 26.25­42.00 |
| A6-L | 37.45 ± 1.007 | 31.50­42.00 | 31.15 ± 0.954 | 21.00­36.75 |
| A6-W | 40.95 ± 1.375 | 31.50­52.50 | 33.95 ± 0.867 | 31.50­42.00 |
| A7-L | 38.50 ± 1.107 | 31.50­42.00 | 32.55 ± 0.760 | 31.50­42.00 |
| A7-W | 46.55 ± 1.838 | 31.50­63.00 | 38.85 ± 1.122 | 31.50­42.00 |
| A8-L | 39.55 ± 1.526 | 31.50­52.50 | 31.15 ± 1.083 | 21.00­36.75 |
| A8-W | 64.75 ± 2.028 | 52.50-84.00 | 57.40 ± 1.658 | 47.25­63.00 |
| A9-L | 56.00 ± 2.028 | 42.00­63.00 | 51.80 ± 1.609 | 42.00­68.25 |
| A9-W | 145.60 ± 3.051 | 126.00­157.50 | 129.15 ± 2.280 | 115.50­147.00 |
| A10-L | 61.60 ± 1.400 | 52.50­73.50 | 52.50 ± 0.887 | 47.25­63.00 |
| A10-W | 169.40 ± 2.482 | 157.50­189.00 | 151.90 ± 3.219 | 126.00­178.50 |
| A11-L | 76.30 ± 3.470 | 52.50­105.00 | 71.75 ± 3.060 | 47.25-89.25 |
| A11-W | 143.50 ± 3.017 | 126.00­168.00 | 127.40 ± 2.318 | 115.50­147.00 |
| C-L | 193.90 ± 3.417 | 168.00­220.50 | 176.05 ± 3.654 | 152.25­199.50 |
| C-W | 169.40 ± 2.482 | 157.50­189.00 | 151.90 ± 3.219 | 126.00­178.50 |
| Total Mt L- blue | 522.20 ± 11.386 | 420.00­577.50 | 452.90 ± 12.544 | 367.50­525.00 |
| Partial Mt L- grey | 340.90 ± 7.590 | 262.50­378.00 | n.a. | n.a. |
| Total Mt L- orange | 476.53 ± 12.999 | 378.00­525.00 | 410.25 ± 10.641 | 346.50­472.50 |
| Partial Mt L- black | 296.42 ± 8.749 | 220.50­346.50 | n.a. | n.a. |
| Proximal Mt W | 82.60 ± 3.816 | 63.00­126.00 | n.a. | n.a. |
| Distal Mt W | 162.40 ± 3.951 | 126.00­189.00 | 134.75 ± 4.023 | 105.00­168.00 |
| Number of Mt spurs | 2.00 ± 0.000 | 2.00­2.00 | 2.00 ± 0.000 | 2.00­2.00 |
| Number of Mt spines | 3.86 ± 0.133 | 2.00­4.00 | 3.86 ± 0.133 | 2.00­4.00 |
| Number of Ms spines | 5.09 ± 0.315 | 4.00-6.00 | 4.90 ± 0.315 | 4.00­6.00 |

Table S2 Means ± SE and ranges of variation of selected morphological characters of Carpophilus truncatus. Abbreviations: L: length; W: width; B: body; P: pronotum; E: elytra; Io: intraocular distance at narrowest point; A1-A11: antennomeres; C: club; Mt: metatibial; Ms: mesotibial; n.a.: not applicable. When not indicated, measures are expressed in µm
