# Supplementary material for: The spread of Carpophilus truncatus is on the razor's edge between an outbreak and a pest invasion
Source: Sci Rep. 2022 Nov 7;12:18841. doi: 10.1038/s41598-022-23520-2 (PMC9640586; doi:10.1038/s41598-022-23520-2)
Supplement: Supplementary file 4 — Supplementary Information 4. [file 41598_2022_23520_MOESM4_ESM.docx]

Table S3 Percentage distribution of qualitative characters of 15 males of Carpophilus truncatus

| **Characters** | **Description** | **Percentage** |
| --- | --- | --- |
| Antenna coloration | Abruptly darker at club | 20% |
|  | Gradually darker towards club | 80% |
| Pronotum setation length on middle of the disc | Long, overlapping adjacent setae | 100% |
|  | Short | 0% |
| Body coloration | Dark brown - black | 94% |
|  | Light brown - brown | 6% |
| Elytra Setation | Evident light setae V-shape | 40% |
|  | Light setae V-shape | 33% |
|  | No setation | 27% |
| Mesotibia shape | Gradually dilated apically | 0% |
|  | Abruptly dilated apically | 100% |
| Metatibia shape | Gradually dilated apically | 0% |
|  | Abruptly dilated apically | 100% |
