# Supplementary material for: The spread of Carpophilus truncatus is on the razor's edge between an outbreak and a pest invasion
Source: Sci Rep. 2022 Nov 7;12:18841. doi: 10.1038/s41598-022-23520-2 (PMC9640586; doi:10.1038/s41598-022-23520-2)
Supplement: Supplementary file 6 — Supplementary Information 6. [file 41598_2022_23520_MOESM6_ESM.docx]

Table S5 List of synonyms of some Carpophilus spp.

| *Carpophilus truncatus* Murray, 1864 (*= C. pilosellus* auct., nec Motschulsky, 1858;  *C. floridanus* Fall, 1910; *C. halli* Dobson, 1954) |
| --- |
| *Carpophilus mutilatus* Erichson, 1843 (= *C. luridus* Murray, 1864;  *C. pilosellus* Motschulsky, 1858) |
