# Supplementary material for: The spread of Carpophilus truncatus is on the razor's edge between an outbreak and a pest invasion
Source: Sci Rep. 2022 Nov 7;12:18841. doi: 10.1038/s41598-022-23520-2 (PMC9640586; doi:10.1038/s41598-022-23520-2)
Supplement: Supplementary file 7 — Supplementary Information 7. [file 41598_2022_23520_MOESM7_ESM.docx]

**Supplementary Table S6** Worldwide distribution of Carpophilus truncatus with the relative references. (*) the species has been reported as Carpophilus pilosellus, (**) as Carpophilus dimidiatus, (***) as Carpophilus halli.

| **Country** | **Reference** |
| --- | --- |
| Argentina** | Reales, N., Rocamundi, N., Marvaldi, A. E., del Carmen Fernández-Górgolas, M., & Stadler, T. (2018). Morphological and molecular identification of *Carpophilus dimidiatus* (Coleoptera: Nitidulidae) associated with stored walnut in Northwestern Argentina. *Journal of Stored Products Research*, 76, 37-42. |
| Australia | Leschen, R. A. B., & Marris, J. W. M. (2005). *Carpophilus* (Coleoptera: Nitidulidae) of New Zealand with notes on Australian species. *Landcare Research Contract Report: LC0405/153*, *35*, 40 |
| Austria | Lobl, I., & Smetana, A. (2007). *Catalogue of Palaearctic Coleoptera* (Vol. 4). Apollo Books. |
| ﻿Bonin Islands | Brown, S. D. J. (2009). Molecular systematics and colour variation of *Carpophilus* species (Coleoptera: Nitidulidae) of the South Pacific. Doctoral dissertation, Lincoln University. |
| ﻿Botswana | Brown, S. D. J. (2009). Molecular systematics and colour variation of *Carpophilus* species (Coleoptera: Nitidulidae) of the South Pacific. Doctoral dissertation, Lincoln University. |
| Caroline Islands* | Gillogly, L. R. (1962). Coleoptera: Nitidulidae. *Insects of Micronesia*, 16(4), 133–188. |
| China | Lobl, I., & Smetana, A. (2007). *Catalogue of Palaearctic Coleoptera* (Vol. 4). Apollo Books. |
| Cook Islands | Brown, S. D. J. (2009). Molecular systematics and colour variation of *Carpophilus* species (Coleoptera: Nitidulidae) of the South Pacific. Doctoral dissertation, Lincoln University. |
| Croatia | Lobl, I., & Smetana, A. (2007). *Catalogue of Palaearctic Coleoptera* (Vol. 4). Apollo Books. |
| Cyprus* | https://fauna-eu.org/cdm_dataportal/taxon/4c3315b0-f866-40e0-a1b1-5c7e8b6456fc |
| ﻿Czech Republic | Lobl, I., & Smetana, A. (2007). *Catalogue of Palaearctic Coleoptera* (Vol. 4). Apollo Books. |
| ﻿Egypt | Atwa, W. A., El-Shaier, M. E. A., & Hala M. K. (2019). Taxonomic Key To the Egyptian Genera and Species of Sap Beetles (Carpophilinae: Nitidulidae: Coleoptera). *Al-Azhar Bulletin of Science*, *30*(2), 1–7. https://doi.org/10.21608/absb.2019.86744 |
| Estonia | Süda, I. (2016). Metsamardikate (Coleoptera) uued liigid Eestis. 2. *Forestry Studies*, *64*, 51–69. https://doi.org/10.1515/fsmu-2016-0004 |
| Fiji | Brown, S. D. J. (2009). Molecular systematics and colour variation of *Carpophilus* species (Coleoptera: Nitidulidae) of the South Pacific. Doctoral dissertation, Lincoln University. |
| France | Lobl, I., & Smetana, A. (2007). *Catalogue of Palaearctic Coleoptera* (Vol. 4). Apollo Books. |
| Germany | Lobl, I., & Smetana, A. (2007). *Catalogue of Palaearctic Coleoptera* (Vol. 4). Apollo Books. |
| Gilbert Islands* | Gillogly, L. R. (1962). Coleoptera: Nitidulidae. *Insects of Micronesia*, 16(4), 133–188. |
| ﻿Greece | Lobl, I., & Smetana, A. (2007). *Catalogue of Palaearctic Coleoptera* (Vol. 4). Apollo Books. |
| Guam* | Gillogly, L. R. (1962). Coleoptera: Nitidulidae. *Insects of Micronesia*, 16(4), 133–188. |
| India | Dasgupta, J., Kumar Pal, T., & Powell, G. S. (2021). Taxonomy of Carpophilinae (Coleoptera: Nitidulidae) from Tripura, India with a New Species. *Annales Zoologici*, *71*(3), 627–649. https://doi.org/10.3161/00034541ANZ2021.71.3.003 |
| Indonesia | Brown, S. D. J. (2009). Molecular systematics and colour variation of *Carpophilus* species (Coleoptera: Nitidulidae) of the South Pacific. Doctoral dissertation, Lincoln University. |
| Iran | Lasoń, A., & Ghahari, H. (2013). A checklist of the Kateretidae and Nitidulidae of Iran (Coleoptera: Cucujoidea). *Zootaxa*, *3746*(1), 101–122. https://doi.org/10.11646/zootaxa.3746.1.4 |
| Italy | Dal Cortivo, M., Sommacal, M., & Gatti, E. (2021). *Chiave dicotomica alle famiglie dei Coleotteri della fauna d’Italia*. |
| Japan | Lasoń, A., & Ghahari, H. (2013). A checklist of the Kateretidae and Nitidulidae of Iran (Coleoptera: Cucujoidea). *Zootaxa*, 3746(1), 101-122. |
| Madagascar | Endrody-Younga, S. (1982). The Nitidulids of the Mascarene Archipelago with additional references to Madagascan species (Coleoptera: Nitidulidae). *Annals of the Transvaaal-Museum*, *33*, 1–180. |
| Mariana Islands* | Gillogly, L. R. (1962). Coleoptera: Nitidulidae. *Insects of Micronesia*, 16(4), 133–188. |
| Marshall Islands* | Gillogly, L. R. (1962). Coleoptera: Nitidulidae. *Insects of Micronesia*, 16(4), 133–188. |
| ﻿Morocco | Brown, S. D. J. (2009). Molecular systematics and colour variation of *Carpophilus* species (Coleoptera: Nitidulidae) of the South Pacific. Doctoral dissertation, Lincoln University. |
| New Zealand | Brown, S. D. J. (2009). Molecular systematics and colour variation of *Carpophilus* species (Coleoptera: Nitidulidae) of the South Pacific. Doctoral dissertation, Lincoln University. |
| Nigeria *** | Dobson, R. M. (1954). A new species of *Carpophilus* Stephens (Col. Nitidulidae) found on stored produce. *Entomologist's Monthly Magazine*, 90, 299-300. |
| Niue | Brown, S. D. J. (2009). Molecular systematics and colour variation of *Carpophilus* species (Coleoptera: Nitidulidae) of the South Pacific. Doctoral dissertation, Lincoln University. |
| Palau* | Gillogly, L. R. (1962). Coleoptera: Nitidulidae. *Insects of Micronesia*, 16(4), 133–188. |
| Papua New Guinea | Brown, S. D. J. (2009). Molecular systematics and colour variation of *Carpophilus* species (Coleoptera: Nitidulidae) of the South Pacific. Doctoral dissertation, Lincoln University. |
| Peru | Cline A. R., Powell G. S., Audisio P. (2015). Beetles (Coleoptera) of Peru: A Survey of the Families. Nitidulidae. *Journal of the Kansas Entomological Society*, *88*(2), 217–220. https://doi.org/10.2317/kent-88-02-173-179.1 |
| Poland | Gawronski R, Lasoń A., & Oleksa A. (2008). Nowe dla Pojezierza Mazurskiego gatunki łyszczynkowatych i ścierowatych (Coleoptera: Nitidulidae, Mycetophagidae). *WIAD. ENTOMOL*, *27*(3), 169–170. https://baza.biomap.pl/en/taxon/species-carpophilus_truncatus/publications |
| Portugal* | https://fauna-eu.org/cdm_dataportal/taxon/4c3315b0-f866-40e0-a1b1-5c7e8b6456fc |
| Republic of Kiribati | Brown, S. D. J. (2009). Molecular systematics and colour variation of *Carpophilus* species (Coleoptera: Nitidulidae) of the South Pacific. Doctoral dissertation, Lincoln University. |
| Republic of Palau | Brown, S. D. J. (2009). Molecular systematics and colour variation of *Carpophilus* species (Coleoptera: Nitidulidae) of the South Pacific. Doctoral dissertation, Lincoln University. |
| Réunion | https://www.gbif.org/occurrence/search?offset=40&taxon_key=6130918 |
| ﻿Seychelles, | Brown, S. D. J. (2009). Molecular systematics and colour variation of *Carpophilus* species (Coleoptera: Nitidulidae) of the South Pacific. Doctoral dissertation, Lincoln University. |
| ﻿Slovakia | Lobl, I., & Smetana, A. (2007). *Catalogue of Palaearctic Coleoptera* (Vol. 4). Apollo Books. |
| Slovenia | Lobl, I., & Smetana, A. (2007). *Catalogue of Palaearctic Coleoptera* (Vol. 4). Apollo Books. |
| Solomon Islands | Brown, S. D. J. (2009). Molecular systematics and colour variation of *Carpophilus* species (Coleoptera: Nitidulidae) of the South Pacific. Doctoral dissertation, Lincoln University. |
| ﻿South Africa | Brown, S. D. J. (2009). Molecular systematics and colour variation of *Carpophilus* species (Coleoptera: Nitidulidae) of the South Pacific. Doctoral dissertation, Lincoln University. |
| Spain* | https://fauna-eu.org/cdm_dataportal/taxon/4c3315b0-f866-40e0-a1b1-5c7e8b6456fc |
| ﻿Sudan | Brown, S. D. J. (2009). Molecular systematics and colour variation of *Carpophilus* species (Coleoptera: Nitidulidae) of the South Pacific. Doctoral dissertation, Lincoln University. |
| Taiwan | Brown, S. D. J. (2009). Molecular systematics and colour variation of *Carpophilus* species (Coleoptera: Nitidulidae) of the South Pacific. Doctoral dissertation, Lincoln University. |
| Turkey | Lasoń, A. (2007). A contribution to the knowledge of the sap beetles of Turkey (Coleoptera: Nitidulidae and Kateretidae). *Annals of the Upper Silesian Museum (Entomology)*, *14*–*15*, 195–221. https://www.researchgate.net/publication/303939096 |
| Tuvalu | Brown, S. D. J. (2009). Molecular systematics and colour variation of *Carpophilus* species (Coleoptera: Nitidulidae) of the South Pacific. Doctoral dissertation, Lincoln University. |
| U.S.A. | Brown, S. D. J. (2009). Molecular systematics and colour variation of *Carpophilus* species (Coleoptera: Nitidulidae) of the South Pacific. Doctoral dissertation, Lincoln University.*; https://www.gbif.org/occurrence/search?offset=40&taxon_key=6130918* |
| Vanuatu | Brown, S. D. J. (2009). Molecular systematics and colour variation of *Carpophilus* species (Coleoptera: Nitidulidae) of the South Pacific. Doctoral dissertation, Lincoln University. |
| West Papua | Brown, S. D. J. (2009). Molecular systematics and colour variation of *Carpophilus* species (Coleoptera: Nitidulidae) of the South Pacific. Doctoral dissertation, Lincoln University. |
